# Two new species of *Semidalis* Enderlein, 1905 (Neuroptera, Coniopterygidae) from China, with an identification key to Chinese species

**DOI:** 10.3897/zookeys.1055.63192

**Published:** 2021-08-05

**Authors:** Yaru Zhao1, Ying Li*, Min Li, Zhiqi Liu

**Affiliations:** 1 Department of Entomology, China Agricultural University, Beijing 100094, China China Agricultural University Beijing China

**Keywords:** Distribution, dustywings, faunistics, lacewings, morphology, taxonomy

## Abstract

Two new species of Coniopterygidae, *Semidalisprocurva***sp. nov.** and *Semidalistibetana***sp. nov.**, are described from China. Both species differ from congeners in characters of the male genitalia. *Semidalisdecipiens* (Roepke, 1916), is recorded from China for the first time. An identification key for adult males of the Chinese species of *Semidalis* Enderlein, 1905 is provided.

## Introduction

**Figure 1.**
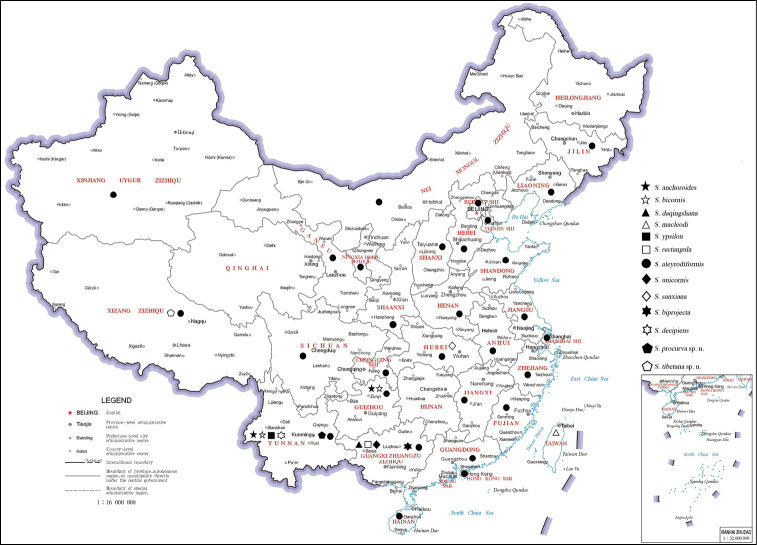
Distribution of *Semidalis* species in China.

The genus *Semidalis* was erected by Enderlein (1905) based on the type species *Semidalisaleyrodiformis* Stephens, 1836 and nowadays possesses approximately 73 species (Sziráki 2011; Oswald 2020). This large genus belongs to the subfamily Coniopteryginae and has a worldwide distribution, with individuals usually found in bushes and trees (Meinander 1972). Both adults and larvae of dustywings are predatory insects, which can feed on spider mites, aphids, scale insects, and plant hoppers (Miller et al. 2004). They are the effective natural enemies in agricultural and forestry production (Abad-Moyano et al. 2009). *Semidalis* is distinguished from other dustywing genera by the combination of the following characters: Rs and M branched in the forewing and hindwing; cross-vein M-Cu1 oblique, striking the branch M_3+4_ or fork of M in both wings; ectoproct and segment 9 synscleritous (Meinander 1972; Aspöck and Aspöck 2008; Sziráki 2011). Up to now, ten species of *Semidalis* have been recorded from China (Sziráki 2011; Oswald 2020). Herein, two new species are described and one species is reported from China for the first time, increasing the number of Chinese *Semidalis* species to thirteen. However, we are aware of two synonyms: *Semidalissanxiana* Liu & Yang, 1997 should be a junior synonym of *Semidalismacleodi* Meinander, 1972 and *Semidalisbiprojecta* Yang & Liu, 1994 should be a junior synonym of *Semidalisanchoroides* Liu & Yang, 1993. These synonyms will be formally proposed in a forthcoming color atlas of the Chinese Neuropterida. So, there should be eleven valid species of *Semidalis* in China. Information on the distribution of *Semidalis* species is shown in Figure 1 and Table 1.

**Table 1.** List of species of the genus *Semidalis* Enderlein, 1905 (Neuroptera, Coniopterygidae) in China.

Species	Distribution(Province)	References
*S.aleyrodiformis* (Stephens, 1836)	Widely distributed	Yang and Liu (1994, 1997); Liu and Yang (1997)
*S.anchoroides* Liu & Yang, 1993	Guizhou, Yunnan	Liu and Yang (1993)
*S.bicornis* Liu & Yang, 1993	Guizhou, Yunnan	Liu and Yang (1993)
*S.biprojecta*^1^ Yang & Liu, 1994	Guangxi	Yang and Liu (1994)
*S.daqingshana* Liu & Yang, 1994	Guangxi	Yang and Liu (1994)
*S.decipiens* (Roepke, 1916)	Yunnan	This paper
*S.macleodi* Meinander, 1972	Taiwan	Meinander (1972); Sziráki, G (2004)
*S.procurva* sp. nov.	Yunnan	This paper
*S.rectangula* Yang & Liu, 1994	Guangxi	Yang and Liu (1994)
*S.sanxiana*^1^ Liu & Yang, 1997	Hubei	Liu and Yang (1997)
*S.tibetana* sp. nov.	Tibet	This paper
*S.unicornis* Meinander, 1972	Guangxi	Meinander (1972); Yang and Liu (1994)
*S.ypsilon* Liu & Yang, 2003	Yunnan	Liu, Yang and Shen (2003)

^1^Species that will be proposed as synonyms (see Introduction).

## Material and methods

Examined specimens are preserved in absolute alcohol and deposited in the Entomological Museum of the China Agricultural University, Beijing (CAU). For the study of genitalic structures, the abdomen was dissected and cleared in a heated solution of 5% potassium hydroxide (KOH) for 5 minutes. Subsequently, the genitalia were rinsed in water and ethanol. Finally, the abdomen was transferred to glycerol for dissection and study. After examination, the abdomen was preserved in glycerol and stored in a 200 μL microtube, while the head and thorax of the specimen were preserved in absolute alcohol and stored in another 200 μL microtube, then the two microtubes were stored in a 5 mL microtube. Morphological terminology follows Meinander (1972). Specimens were examined with an Optec SZ760 stereomicroscope. Photos were taken with a Nikon D5300 digital camera attached to a Leica DM2500 stereomicroscope. The resulting images were edited and processed with Adobe Photoshop CC 2018. According to the results of the photos and the observation under the microscope, the pen and pencil tools of the Photoshop software were used to draw the various views of the genitals, then the photos and drawings were typeset on the software to generate the final picture.

## Taxonomy

### Genus *Semidalis* Enderlein, 1905

#### Semidalis decipiens

Taxon classificationAnimaliaNeuropteraConiopterygidae

(Roepke, 1916)

FFB9CD73-076A-5779-9BBA-632673EE367F

Figs 2 , 3

##### Type species.

*Semidalisaleyrodiformis* Stephens, 1836.

##### Material examined.

China: Yunnan (Province): Ruili (County): Longchuan (Township), [24.1776°N, 97.7947°E], 28.iii.2019, leg. Yaru Zhao and Mingming Zou, 70 males. China: Yunnan (Province): Jinghong (City), [21.9695°N, 100.8060°E], 23.iii.2019, leg. Yaru Zhao and Mingming Zou, 7 males. China: Yunnan (Province): Ruili (County): Guangshuang (Village), [23.9500°N, 97.7880°E], 1.v.1981, leg. Fasheng Li, 1 male. China: Yunnan (Province): Ruili (County): Longchuan (Township), [24.1776°N, 97.7947°E], 1.v.1981, leg. Chikun Yang, 2 males. China: Yunnan (Province): Menghai (County), [22.0031°N, 100.2050°E], 9.iv.1981, leg. Fasheng Li, 1 male. China: Yunnan (Province): Jinghong (City), [22.0285°N, 100.9025°E], 9.iv.1981, leg. Fasheng Li, 1 male.

##### Measurements.

**Male**: Body length 1.8–2.4 mm. Antennae 31–34 segment, 2.1–2.3 mm in length. Forewing length 2.2–3.0 mm, width 1.0–1.1 mm. Hindwing length 2.0–2.4 mm, width 0.8–0.9 mm (*N* = 20).

##### Redescription.

**Figure 2.**
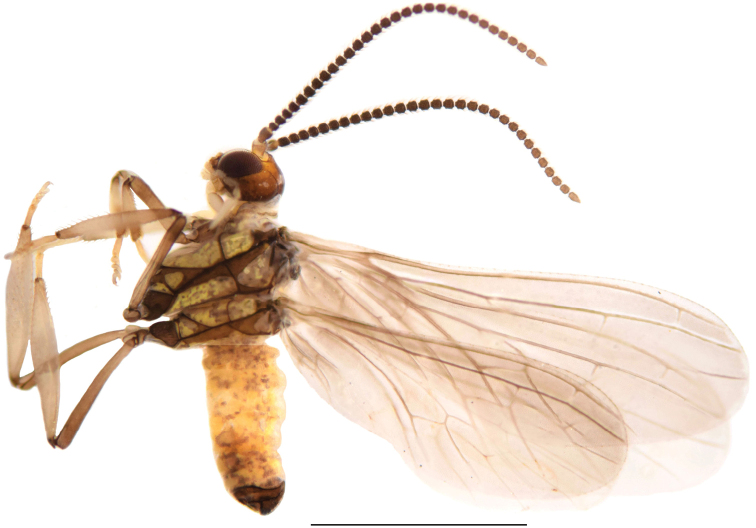
*Semidalisdecipiens* (Roepke, 1916), male habitus, lateral view. Scale bar: 1 mm.

***Head*** (Fig. 2). Dark brown. Frons and palpi normal. Compound eyes large and dark. Antennae brown. Scape and pedicel broad and blunt. Basal flagellomeres wider than long. Distal flagellomeres gradually tapering toward apex. Apical flagellomeres tapered. Pedicel and flagellomeres each with two circles of hair-like sensilla. Maxillary and labial palps yellowish brown.

***Thorax*** (Fig. 2). Pale ochreous. Thoracic apodemes light brown. Sutures dark brown. Meso- and metanotum with shoulder spots. Legs brown.

***Wing*.** Wing membrane light greyish brown, almost hyaline.

**Figure 3.**
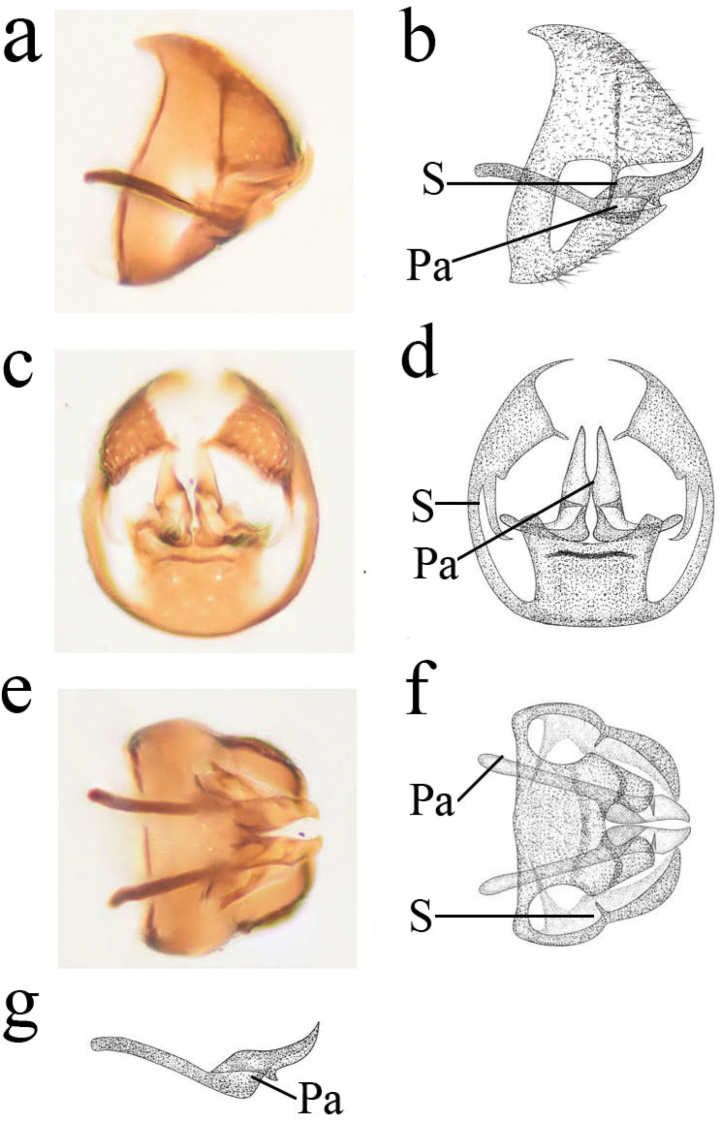
Semidalisdecipiens (Roepke, 1916), male genitalia **a, b** genitalia, lateral view **c, d** genitalia, caudal view **e, f** genitalia, ventral view **g** parameres, lateral view. Pa, parameres; S, stylus.

***Male terminalia*** (Fig. 3). Abdomen pale ochreous. Segment 9 wholly synscleritous, proximally strengthened by an apodeme encircling the whole abdomen. Ectoproct subtriangular in lateral view; dorso-caudal angle acute in caudal view. Stylus slender and apparently projecting from the border of ectoprocts and segment 9. Hypandrium truncate apically in lateral view; dorsal margin with a shallow incision in caudal view. Parameres pick-like; basal part slender; distal part widened and curved upwards decidedly, with ventral knob in middle part. Uncini absent.

##### Remarks.

This species is reported from China for the first time.

##### Distribution.

China (new record), Yunnan; India; Indonesia; Malaysia (Sziráki 2011).

#### Semidalis procurva

Taxon classificationAnimaliaNeuropteraConiopterygidae

Zhao, Y. Li, M. Li & Liu sp. nov.

588FCBDA-8EDF-5C79-BFDF-0A98DE85B3DC

http://zoobank.org/79B319E3-01AD-4EFA-B044-2E2629262EBC

Figs 4 , 5

##### Type material.

***Holotype*** male, China: Yunnan (Province): Ruili (County): Mengxiu (Township): Nanjingli (Village), [24.0917°N, 97.8460°E], 30.iii.2019, leg. Yaru Zhao. ***Paratypes*.** Same data as holotype, 37 males. CHINA: Yunnan (Province): Ruili (County): Mengxiu (Township): Nanjingli (Village), [24.0917°N, 97.8460°E], 2.v.1981, leg. Chikun Yang, 3 males.

##### Diagnosis.

**Male genitalia**: stylus present; parameres with ventral knob, long, distal part widened and bent upwards, and apical part bent forwards distally in an obtuse angle; uncini absent.

##### Measurements.

**Male**: Forewing length 3.2–3.5 mm, width 1.1–1.3 mm. Hindwing length 2.5–2.7 mm, width 1.0–1.3 mm. Body length 2.4–2.6 mm. Antennae 34–35 segments, 2.1–2.3 mm in length (*N* = 15).

##### Description.

**Figure 4.**
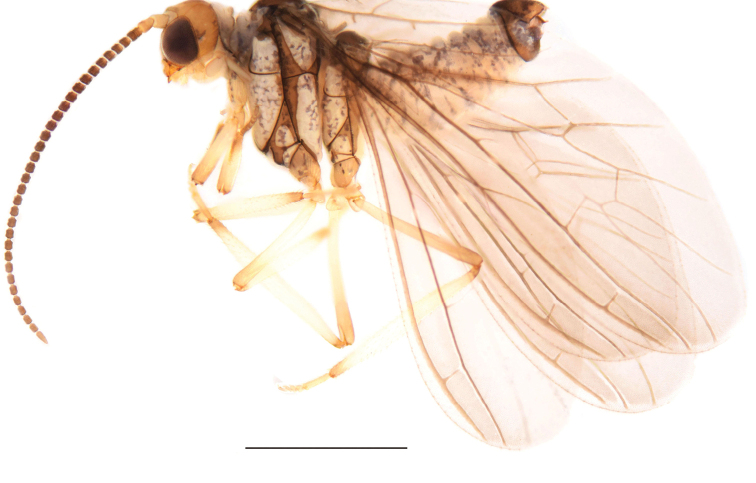
*Semidalisprocurva* sp. nov., male habitus, lateral view. Scale bar: 1 mm.

**Male: *Head*** (Fig. 4). Brown. Frons and palpi normal. Compound eyes large, dark. Scape and pedicel broad, blunt and yellowish brown. Flagellomeres dark brown. Basal flagellomeres as long as broad. Subsequent flagellomeres tapering gradually. Apical flagellomeres tapered. Flagellomeres each with two circles of hair-like sensilla. Maxillary and labial palps yellowish brown.

***Thorax*** (Fig. 4). Pale ochreous. Thoracic apodemes light brown. Sutures dark brown. Meso- and metanotum with shoulder spots. Legs brown (except femur and tibia yellowish brown).

***Wing*.** Wing membrane yellowish brown, almost hyaline.

**Figure 5.**
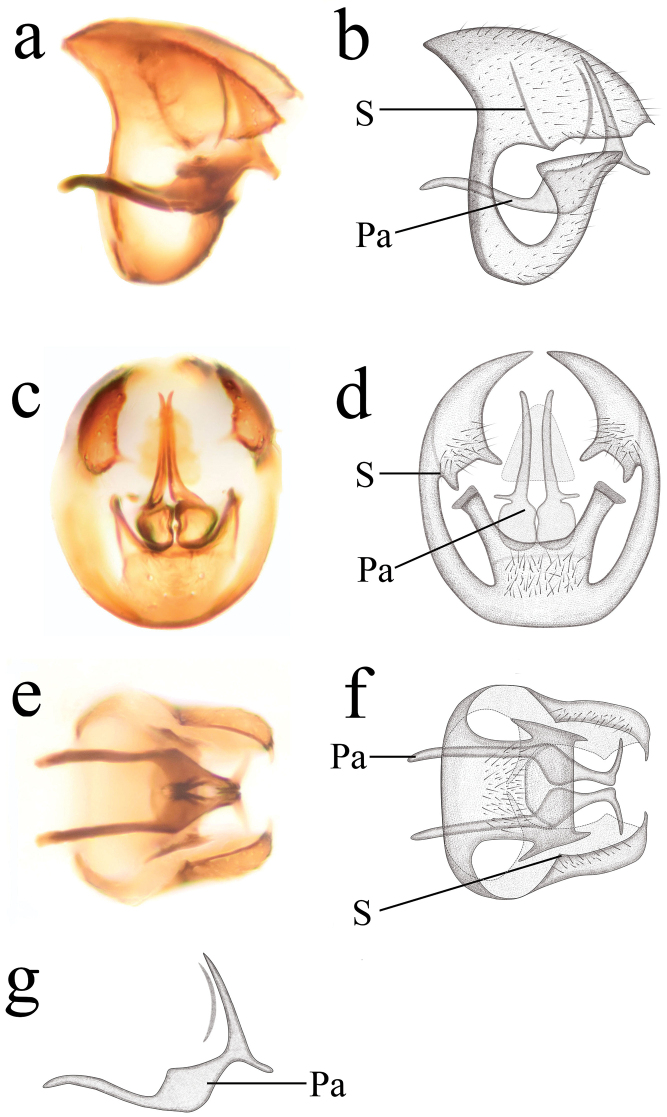
Semidalisprocurva sp. nov., male genitalia **a, b** genitalia, lateral view **c, d** genitalia, caudal view **e, f** genitalia, ventral view **g** parameres, lateral view. Pa, parameres; S, stylus.

***Male terminalia*** (Fig. 5). Abdomen pale ochreous. Segment 9 wholly synscleritous, proximally strengthened by an apodeme encircling the whole abdomen. Ectoproct subtriangular in lateral view; dorso-caudal angle acute in caudal view. Stylus small at the border of ectoprocts and segment 9. Hypandrium truncate apically in lateral view; dorsal margin with a deep incision in caudal view; lateral process slender in caudal view. Parameres long; basal part slender; distal part widened and bent upwards, apical part bent forwards in an obtuse angle, with ventral knob, connected with a small sclerite. Uncini absent.

##### Distribution.

China (Yunnan).

##### Etymology.

The species name is a Latin adjective referring to the forward bending pose on distal part of parameres.

##### Remarks.

The new species belongs to the *Semidalisrectangular* group. It is similar to *Semidalismacleodi* Meinander, 1972, but the two species differ in the shape of parameres. The apical part of the parameres is bent forwards in an obtuse angle in the new species, while it is bent upwards in *S.macleodi*. Moreover, a ventral knob is present in the proximal two thirds of the parameres in the new species, while it is present at the tip of the parameres in *S.macleodi*.

#### Semidalis tibetana

Taxon classificationAnimaliaNeuropteraConiopterygidae

Zhao, Y. Li, M. Li & Liu sp. nov.

D7D41DAF-F8B1-5EE6-80F3-2D7F8E2A76EB

http://zoobank.org/7B69881E-8AB3-46C0-BFD9-4A4DD01BF02C

Figs 6 , 7

##### Type material.

***Holotype*** male, China: Tibet (Autonomous Region): Linzhi (City): Milin (County), [29.0428°N, 93.8898°E], 10.vi.2019, leg. Yaru Zhao. ***Paratypes*.** Same data as holotype, 37 males.

##### Other material.

China: Tibet (Autonomous Region): Linzhi (City): Bomi (County): Zhamu (Township), [29.7103°N, 95.5857°E], 10–19.vi.1978, leg. Fasheng Li, 92 males. China: Tibet (Autonomous Region): Linzhi (City): Linzhi (County), [29.6019°N, 94.4168°E], 7.vi.1978, leg. Fasheng Li, 1 male. China: Tibet (Province): Linzhi (City): Bomi (County): Yigong (Township), [30.2389°N, 94.8523°E], 14.vi.1978, leg. Fasheng Li, 2 males. China: Tibet (Autonomous Region): Linzhi (City): Chayu (County), [29.7103°N, 95.5857°E], 2.vi.1978, leg. Fasheng Li, 4 males.

##### Diagnosis.

**Male genitalia**: stylus present; parameres without ventral knob, distal part gradually widened and bent upwards in an obtuse angle, apical part conspicuously bent forwards distally; uncini absent.

##### Measurements.

**Male**: Forewing length 2.6–3.5 mm, width 1.5–1.9 mm. Hindwing length 2.1–2.8 mm, width 1.2–1.5 mm. Body length 1.6–2.3 mm. Antennae 33–35 segments, 2.3–2.5 mm in length (*N* = 25).

##### Description.

**Figure 6.**
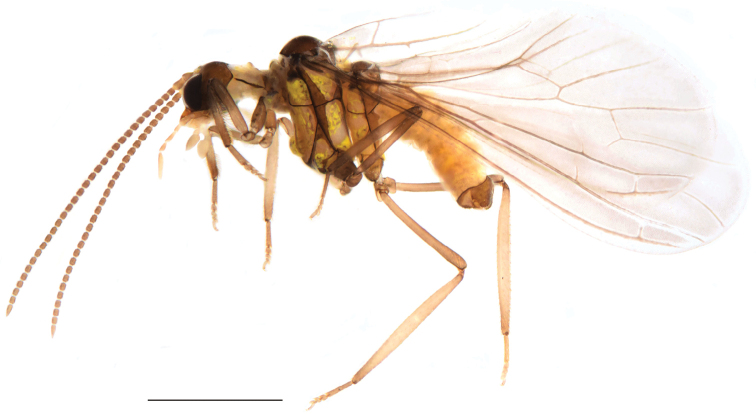
*Semidalistibetana* sp. nov., male habitus, lateral view. Scale bar: 1 mm.

**Male: *Head*** (Fig. 6). Dark brown. Frons and palpi normal. Compound eyes large and dark. Antennae brown. Scape and pedicel broad and blunt. Basal flagellomeres as long as wide. Distal flagellomeres gradually tapering toward apex. Apical flagellomeres tapered. Pedicel and flagellomeres each with two circles of hair-like sensilla. Maxillary and labial palps light brown.

***Thorax*** (Fig. 6). Pale ochreous. Thoracic apodemes light brown. Sutures dark brown. Meso- and metanotum with shoulder spots. Legs light brown.

***Wing*.** Wing membrane dark brown, almost hyaline.

**Figure 7.**
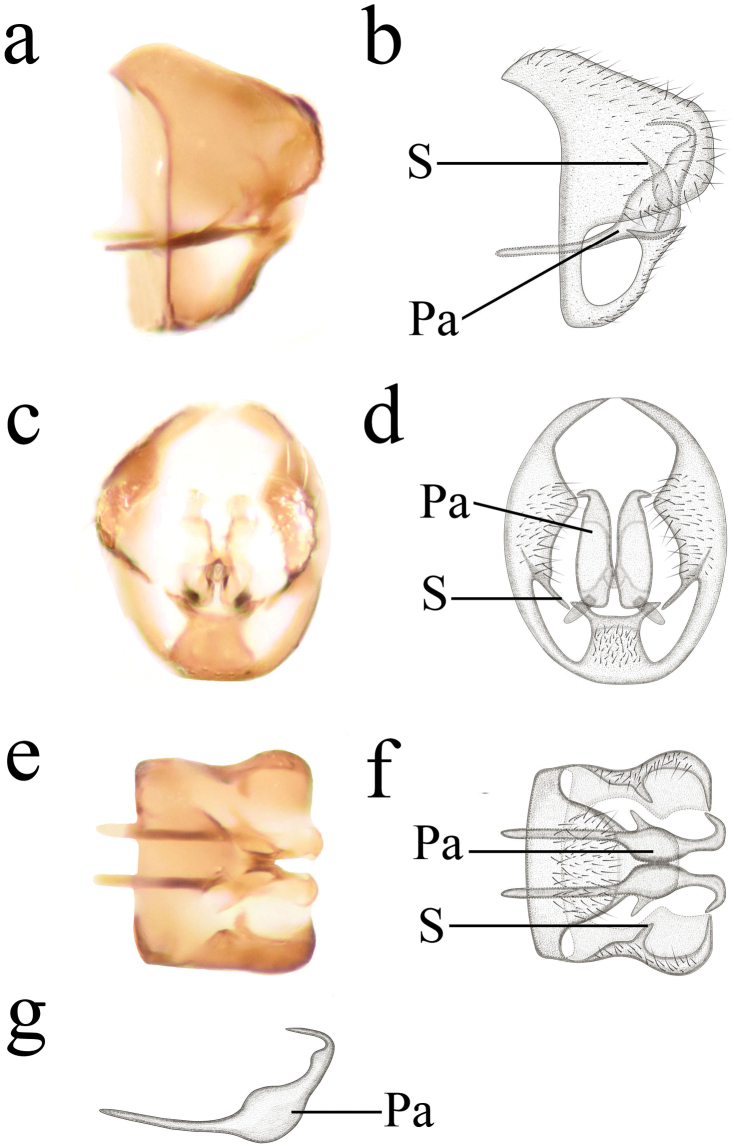
Semidalistibetana sp. nov., male genitalia **a, b** genitalia, lateral view **c, d** genitalia, caudal view. **e, f** genitalia, ventral view **g** parameres, lateral view. Pa, parameres; S, stylus.

***Male terminalia*** (Fig. 7). Abdomen pale ochreous. Segment 9 wholly synscleritous, proximally strengthened by an apodeme encircling the whole abdomen. Ectoproct short, broad and round. Hypandrium truncate apically in lateral view; dorsal margin with a deep incision in caudal view; lateral process slender in caudal view. Stylus long and broad, apparently projecting from the border of ectoprocts and segment 9. Parameres long; basal part slender; distal part gradually widened and bent upwards in an obtuse angle, and its apical part bent forwards evidently. Uncini absent.

##### Distribution.

China (Tibet).

##### Etymology.

The specific epithet “*tibetana*” refers to the name of type locality.

##### Remarks.

The new species belongs to the *Semidalisrectangular* group. It is similar to *Semidalisrectangular* Yang & Liu, 1994, but the two species differ in the shape of the parameres. The distal part of the parameres is bent upwards in an obtuse angle in the new species, while it is bent upwards clearly in *S.rectangular*. Moreover, the apical part of the parameres bends forward distinctly in the new species, while it is bent upwards in *S.rectangular*. Furthermore, the stylus is long and broad in the new species, while it is small in *S.rectangular*.

### Identification key to males of Chinese species of *Semidalis*

**Table:** 

1	Parameres with ventral knob (Figs 3g, 5g)	**2**
–	Parameres without ventral knob (Fig. 7g)	**5**
2	Ventral knob at distal part of parameres (Meinander 1972: fig. 198B)	***S.macleodi* Meinander**
–	Ventral knob at proximal two thirds of parameres (Figs 3g, 5g)	**3**
3	Parameres with uncinus (Liu et al. 2003: figs 4–8)	***S.ypsilon* Liu & Yang**
–	Parameres without uncinus (Figs 3g, 5g)	**4**
4	Ventral knob small in parameres (Fig. 3g)	***S.decipiens* (Roepke)**
–	Ventral knob slender in parameres (Fig. 5g)	***S.procurva* Zhao, Y. Li, M. Li & Liu, sp. nov.**
5	Uncini absent	**6**
–	Uncini present (Meinander 1972: fig. 200F)	**9**
6	Tip of parameres bent forwards (Fig. 7g)	***S.tibetana* Zhao, Y. Li, M. Li & Liu, sp. nov.**
–	Tip of parameres bent upwards (Yang and Liu 1994: fig. 5)	***S.rectangula* Yang & Liu**
7	Uncini fused (Meinander 1972: fig. 212E)	**8**
–	Uncini not fused	**9**
8	Distal part of hypandrium composing one spine in caudal view (Meinander 1972: fig. 212F)	***S.unicornis* Meinander**
–	Distal part of hypandrium composing two spines in caudal view (Liu and Yang 1993: fig. 4C)	***S.bicornis* Liu & Yang**
9	Parameres with one dorsal knob (Liu and Yang 1993, fig. 3C)	***S.anchoroides* Liu & Yang**
–	Parameres with two dorsal knobs (Meinander 1972: fig. 200F)	**10**
10	Uncini present near the middle part of parameres (Meinander 1972: fig. 200F)	***S.aleyrodiformis* (Stephens)**
–	Uncini present at the distal part of parameres (Yang and Liu 1994: fig. 4)	***S.daqingshana* Liu & Yang**

## Supplementary Material

XML Treatment for Semidalis decipiens

XML Treatment for Semidalis procurva

XML Treatment for Semidalis tibetana
